# Supplementary material for: Expression of CD20 after viral reactivation renders HIV-reservoir cells susceptible to Rituximab
Source: Nat Commun. 2019 Aug 16;10:3705. doi: 10.1038/s41467-019-11556-4 (PMC6697690; doi:10.1038/s41467-019-11556-4)
Supplement: Supplementary file 1 — Supplementary Information [file 41467_2019_11556_MOESM1_ESM.pdf]

SUPPLEMENTARY INFORMATION

Expression of CD20 after Viral Reactivation Renders HIV-Reservoir Cells Susceptible to Rituximab

Carla Serra-Peinado, Judith Grau-Expósito, Laura Luque-Ballesteros, Antonio Astorga-Gamaza, Jordi Navarro, Jenny Gallego-Rodriguez, Mario Martin, Adrià Curran, Joaquin Burgos, Esteban Ribera, Berta Raventós, Rein Willekens, Ariadna Torrella, Bibiana Planas, Rosa Badía, Felipe Garcia, Josep Castellví, Meritxell Genescà, Vicenç Falcó, and Maria J. Buzon

Table S1. Clinical data of patients included in the study.

Patient ID (new)	Time since HIV diagnosis (months)	CD4 Cell Count (cells/µl)	%CD4	Viral Load (copies/ml)	Time on ART (months)	ART regimen
**1**	300	580	20.19	<50	122	DRV/c
**2**	50	1,120	40.2	<50	28	TDF+FTC+DRV/r
**3**	11	280	22.45	<50	4	ABC+3TC+DTG
**4**	25	1,030	34.87	<50	16	TDF+FTC+EVG/c
**5**	146	1,830	51.37	<50	48	EFV+TDF+3TC
**6**	267	1,020	55.21	<50	120	FTC+NVP+TDF
**7**	166	1,180	40.85	<50	88	FTC+RPV+TDF
**8**	245	1,060	-	<50	179	FTC+RPV+TDF
**9**	66	990	45.96	<50	21	TDF+FTC+RPV
**10**	36	1,150	47.33	<50	19	TDF+FTC+RPV
**11**	240	150	-	<50	156	c/DRV
**12**	247	1,050	32.03	<50	206	c/DRV
**13**	216	1,150	38.31	<50	170	c/DRV
**14**	298	1,200	-	<50	176	COB/DRV
**15**	10	680	39.7	<50	10	ABC+3TC+RPV
**16**	32	280	22	<50	27	TDF+FTC+RPV
**17**	43	790	35.4	<50	21	TDF+FTC+EVG/c
**18**	48	800	40.42	<50	23	ABC+3TC+RAL
**19**	46	2,290	27.8	<50	25	ABC+3TC+RPV
**20**	31	1,230	55.8	<50	26	ABC+3TC+RPV
**21**	279	330	15.98	<50	18	TDF+FTC+EVG/c
**22**	30	130	7.38	<50	25	ABC+3TC+RAL
**23**	30	1,290	61.79	<50	23	TDF+FTC+RPV
**24**	40	530	27	<50	21	TDF+FTC+RPV
**25**	56	990	42.56	<50	33	TDF+FTC+DRV/r
**26**	47	490	36.57	<50	18	ABC+3TC+RPV
**27**	78	1,210	45.93	<50	29	TDF+FTC+EVG/c
**28**	153	770	49.03	<50	51	EFV+FTC+TDF
**29**	292	560	-	<50	156	COB+DRV+RTG
**30**	340	1,040	-	<50	168	DRV+FTC+RTV+TDF
**31**	94	600	10.3	<50	40	DRV+RTV
**32**	115	360	30	<50	61	LPV+RTV
**33**	72	800	-	<50	18	3TC+ABV+DTG
**34**	33	490	-	<50	28	EVG+FTC+TAF/c
**35**	31	560	-	<50	11	EVG+FTC+TAF/c
**36**	49	1,070	-	<50	40	EFV+FTC+TDF
**37**	31	810	-	<50	22	3TC+ABV+DTG
**38**	42	1,150	-	<50	37	3TC+ABV+RPV
**39**	13	1,760	-	<50	13	EVG+FTC+TAF/c
**40**	55	970	-	<50	45	3TC+ABC+DTG
**41**	28	910	33.97	<50	22	TDF+FTC+EVG/c
**42**	17	750	35.79	<50	16	ABC+3TC+RPV
**43**	24	-	-	<50	23	TDF+FTC+EFV

**44**	25	600	31.23	<50	17	ABC+3TC+ATV/r
**45**	132	740	-	<50	79	3TC+ABC+DTG
**46**	136	710	-	<50	115	3TC+ABC+DTG
**47**	106	810	-	<50	101	3TC+ABC+RPV
**48**	310	340	-	<50	79	DRV+FTC+TDF/c
**49**	59	800	-	<50	41	FTC+RPV+TDF
**50**	66	1,130	-	<50	19	EVG+FTC+TAF/c
**51**	42	430	-	<50	38	EVG+FTC+TAF/c
**52**	300	2,020	-	<50	-	3TC+ABV+DTG
**53**	129	270	-	<50	126	3TC+DTG
**54**	319	689	-	<50	136	DRV+ETV+RTG/r
**55**	111	600	-	<50	105	FTC+RPV+TDF
**56**	1042	1,010	24.7	<50	624	3TC+ABV+DTG
**57**	750	180	18.75	<50	112	3TC+ABC+DTG
**58**	306	1,080	39.5	<50	273	3TC+ABV+RPV
**59**	67	715	39.72	<50	66	FTC+RPV+TDF
**60**	8	420	30.46	74,500	-	UNT
**61**	72	320	22.09	47,300	-	UNT
**62**	0	570	26.41	60,300	-	UNT
**63**	2	600	24.69	296,000	-	UNT
**64**	84	330	8.89	79,200	-	UNT
**65**	0	610	36.88	119,000	-	UNT
**66**	0	480	18.27	178,000	-	UNT
**67**	0	960	37.03	145,000	-	UNT
**68**	0	920	41.2	9,560	-	UNT
**69**	226	240	9.65	272	-	UNT
**70**	0	430	23.2	3,850,000	-	UNT
**71**	0	480	26.27	12,800,000	-	UNT
**72**	3	190	38.82	23,300,000	-	UNT
**73**	67	790	35.43	20,300	-	UNT
**74**	44	760	21.09	11,000	-	UNT
**75**	36	760	30.17	22,700	-	UNT
**76**	218	360	19.79	880	-	UNT
**77**	197	170	6.93	517,000	-	UNT
**78**	205	280	18.25	250,000	-	UNT
**79**	2	180	4.36	405,000	-	UNT
**80**	0	780	27.1	302,000	-	UNT
**81**	1	550	42.71	92	-	UNT
**82**	0,1	150	7.5	5,000,000	-	UNT
**83**	85	314	17	56,000	-	UNT
**84**	88	368	20.5	56,000	-	UNT
**85**	51	407	21.04	14,000	-	UNT
**86**	0	310	-	80,400	-	UNT

FTC, emtricitabine; TDF, tenofovir; NVP, nevirapine; ATV, atazanavir; 3TC, lamivudine; EFV, efavirenz; ABC, abacavir; RAL, raltegravir; EVG, elvitegravir; DTG, dolutegravir; DRV, darunavir; RPV, Rilpivirine; TAF, tenofovir alafenamida; LPV, Lopinavir; /r, boosted with ritonavir; /c, boosted with cobicistat; UNT, untreated.

Supplementary Figure 1

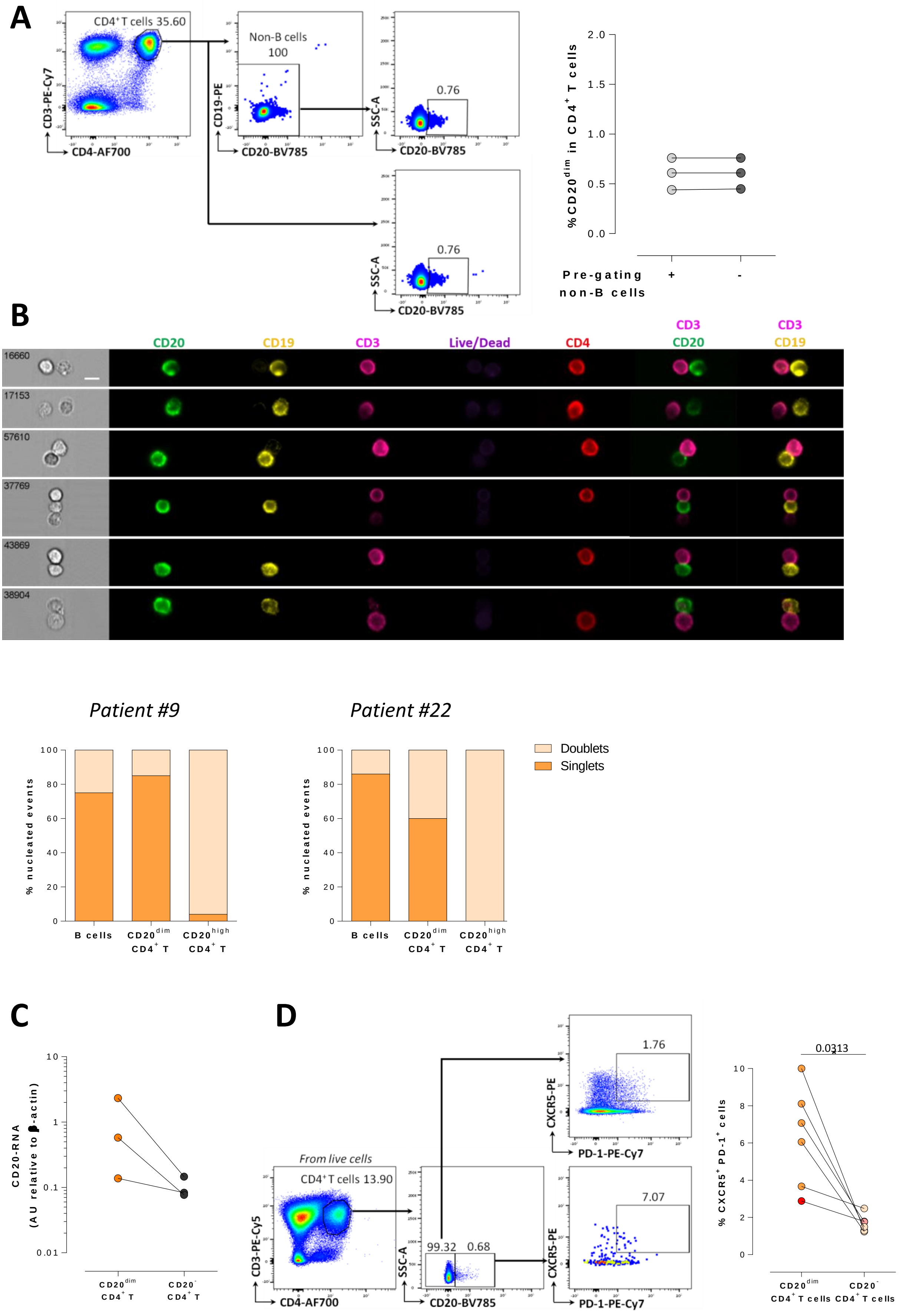

Supplementary Figure 1. Characterization of CD20^{dim} CD4^{+}T cell subpopulation. A. Left, gating strategy used to identify CD20^{dim} cells, with and without inclusion of the CD19 marker. Previous sequential gates are represented in Supplementary Figure 9. Right, comparison of percentages of the CD20^{dim} CD4^{+} T cell population with or without B cell exclusion in n=3 ART-suppressed patients. **B.** Representative bright-field and color fluorescence images of T-B cells conjugates found in the CD20^{high} CD4^{+} T cell population of ART-suppressed patients (#9 and 22). Percentage of singlet (dark orange) and doublet (light orange) events among total nucleated events detected in each population. Individual graphs from each ART-suppressed patients. Scale bar 10 µm. **C.** CD20-mRNA was measured in sorted CD20^{dim} CD4^{+}T cells (orange) and CD20^{-} CD4^{+}T cells (black). Relative expression of CD20-mRNA to β-actin-mRNA of n=3 ART-suppressed patients (#49-51), arbitrary units (AU). **D.** Characterization of follicular helper cells (T_{FH}) phenotype defined by CXCR5 and PD-1 expression in CD20^{-} or CD20^{dim} CD4^{+} T cells**.** Left, gating strategy used to determine CXCR5 and PD-1 expression. Right, expression of CXCR5 and PD-1 in CD20^{dim} and CD20^{-} CD4^{+} T cells in n=5 ART-suppressed (#17-19, 54 and 55, orange) and n=1 viremic (#86, red) patients. Wilcoxon comparison test was performed. Data underlying this Figure is provided as Source Data file.

Supplementary Figure 2

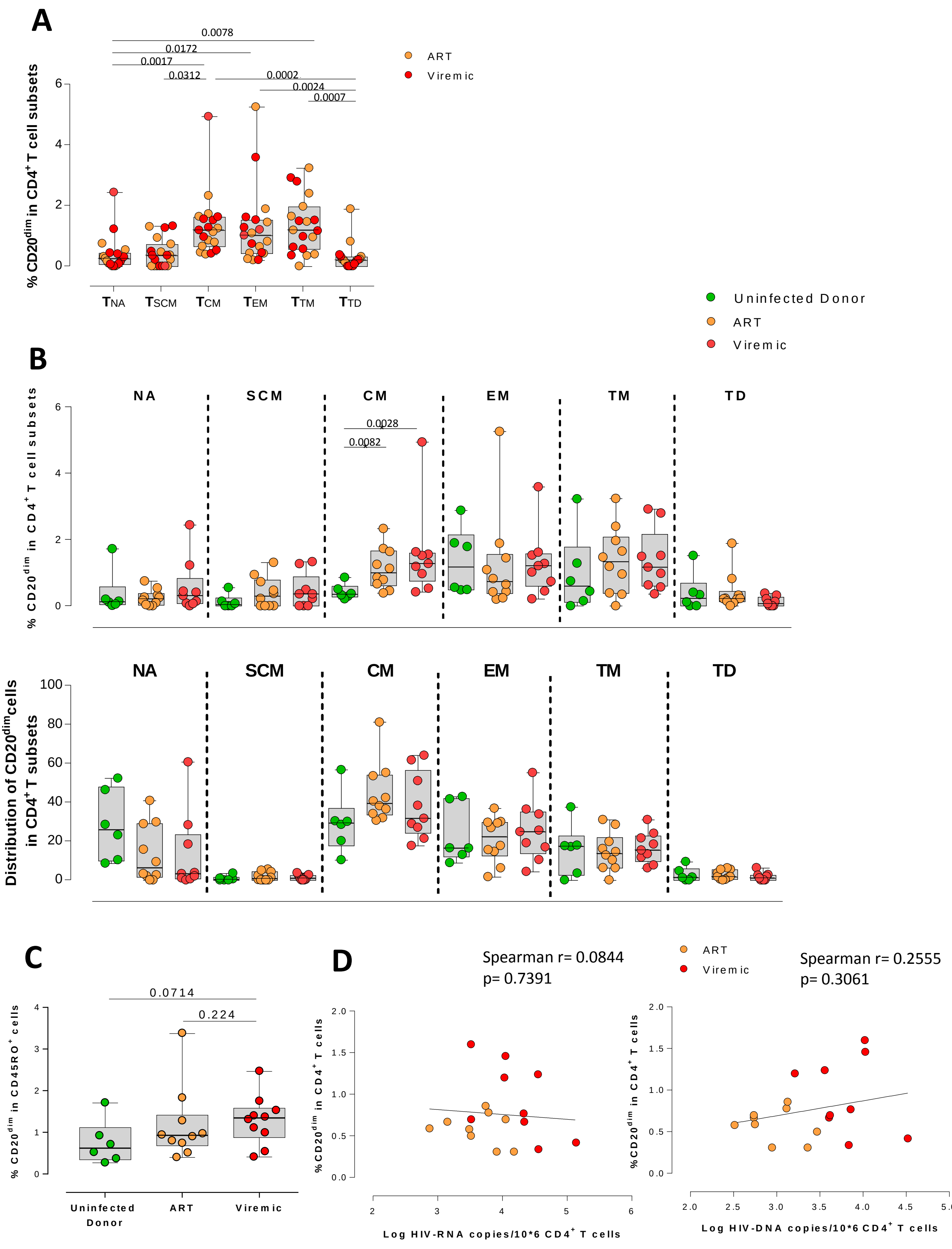

Supplementary Figure 2. Expression and distribution of CD20^{dim} cells in CD4^{+} T cell subsets. **A.** Frequency of CD20^{dim} cells in T cell subsets of viremic (red) and ART-suppressed (orange) individuals. ANOVA and Dunn's multiple comparison test were performed. **B.** The upper panel shows percentages of expression of CD20^{dim} in CD4^{+} T cell subsets. The lower panel shows the distribution of CD20^{dim}-expressing cells in different CD4^{+} T cell subsets. In both panels, the median of uninfected donors, HIV^{+} ART-suppressed patients and HIV^{+} viremic patients is presented. Mann-Whitney test. In panels A and B, patients #1-10, 60-65, 67-69 are represented, n=6 HIV^{-} donors, n=10 ART-suppressed patients, n=9 viremic patients. **C.** Percentage of CD4^{+} T memory subsets (defined as CD45RO^{+} cells) expressing CD20^{dim} in different cohorts of patients. Panels A-C median and min-max rank are represented. **D.** Correlation of CD20^{dim} CD4^{+} T cells with intracellular HIV RNA (left) and total HIV DNA (right) are shown. Data from n=9 ART-suppressed (#2-10, orange) and n=9 viremic (#60-65, 67-69, red) patients are included. Spearman's nonparametric correlation coefficients and associated *P* values are shown. Gating strategy used for these analyses is shown in Figure 1A. Data underlying this Figure is provided as Source Data file.

Supplementary Figure 3

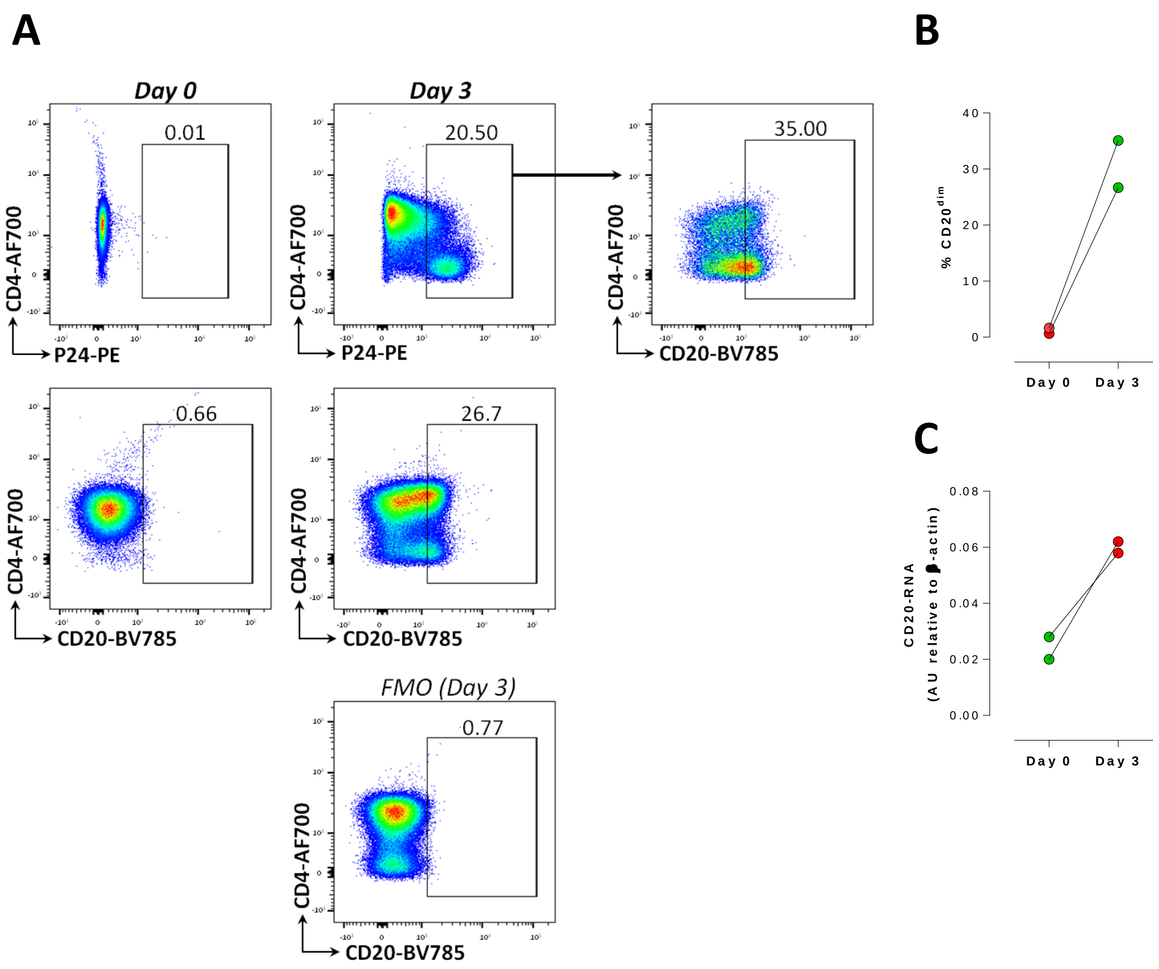

Supplementary Figure 3. Upregulation of CD20 expression after ex vivo HIV infection in previously isolated CD20⁻ CD4⁺ cells. CD4⁺ T cells non-expressing CD20 were isolated by cell sorting. The next day, cells were infected with the NL4.3 viral strain. CD20 expression was measured by flow cytometry and mRNA quantification at day 0 and 3 post-infection. **A**. Gaiting strategy used to identify p24⁺ cells (upper panel) and CD20 expression (lower panel). Previous sequential gates are represented in Supplementary Figure 9. **B.** Percentages of cells expressing CD20 in CD4⁺ T cells before and after infection. **C.** CD20-RNA levels after ex vivo infection. Relative expression of CD20-mRNA to β-actin-mRNA, arbitrary units (AU), from n=2 ex vivo infected cells. Data underlying this Figure is provided as Source Data file.

Supplementary Figure 4

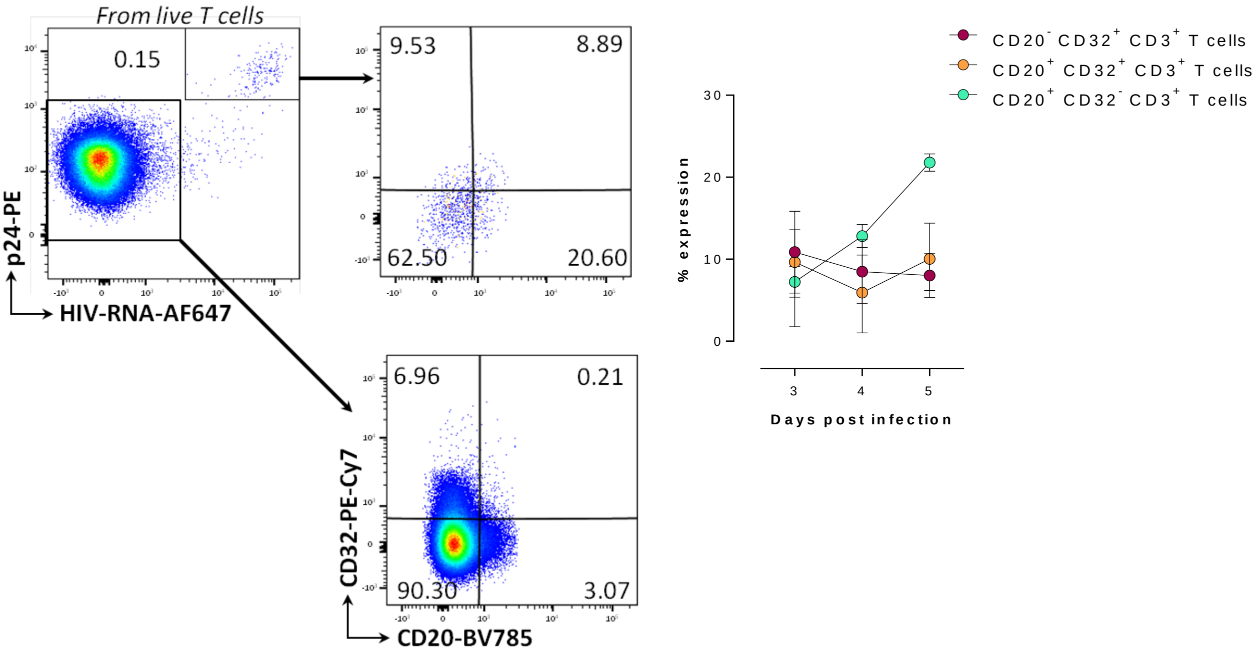

Supplementary Figure 4. Expression of CD20 and CD32 in ex vivo-infected cells. Unstimulated PBMCs from three uninfected donors were infected with the HIV strain NL4.3. Infection was monitored by the simultaneous staining of HIV RNA using the RNA FISH-flow assay and the viral protein p24 at days 3, 4 and 5 after infection. The left panel shows the gating strategy used to monitor HIV infection and expression of CD20 and CD32. Previous sequential gates are represented in Supplementary Figure 9. The right panel shows the percentage of productively HIV-infected cells with CD20, CD32, and double-positive expression. Mean and SEM are presented from 3 independent experiments. Data underlying this Figure is provided as Source Data file.

Supplementary Figure 5

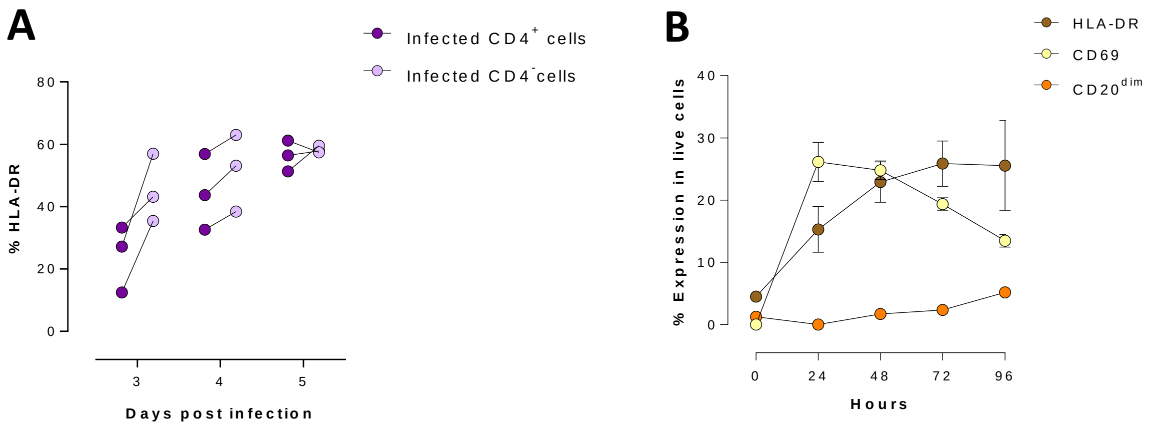

Supplementary Figure 5. Expression of HLA-DR in ex vivo-infected cells and expression of CD20 during activation of CD4^{+} T cells. A. Frequency of HLA-DR in HIV-infected cells at days 3, 4 and 5 after ex vivo infection of unstimulated PBMCs from three uninfected donors. **B.** Frequency of CD20^{dim}, HLA-DR and CD69 in PBMCs from uninfected donors after stimulation with anti-CD3 (1 µg/ml) and anti-CD28 (2 µg/ml) for 96 h. Mean and SD are presented from 3 independent experiments for HLA-DR and PD-1, and one experiment for CD20. Flow gating strategy is shown in Figure 5. Data underlying this Figure is provided as Source Data file.

Supplementary Figure 6

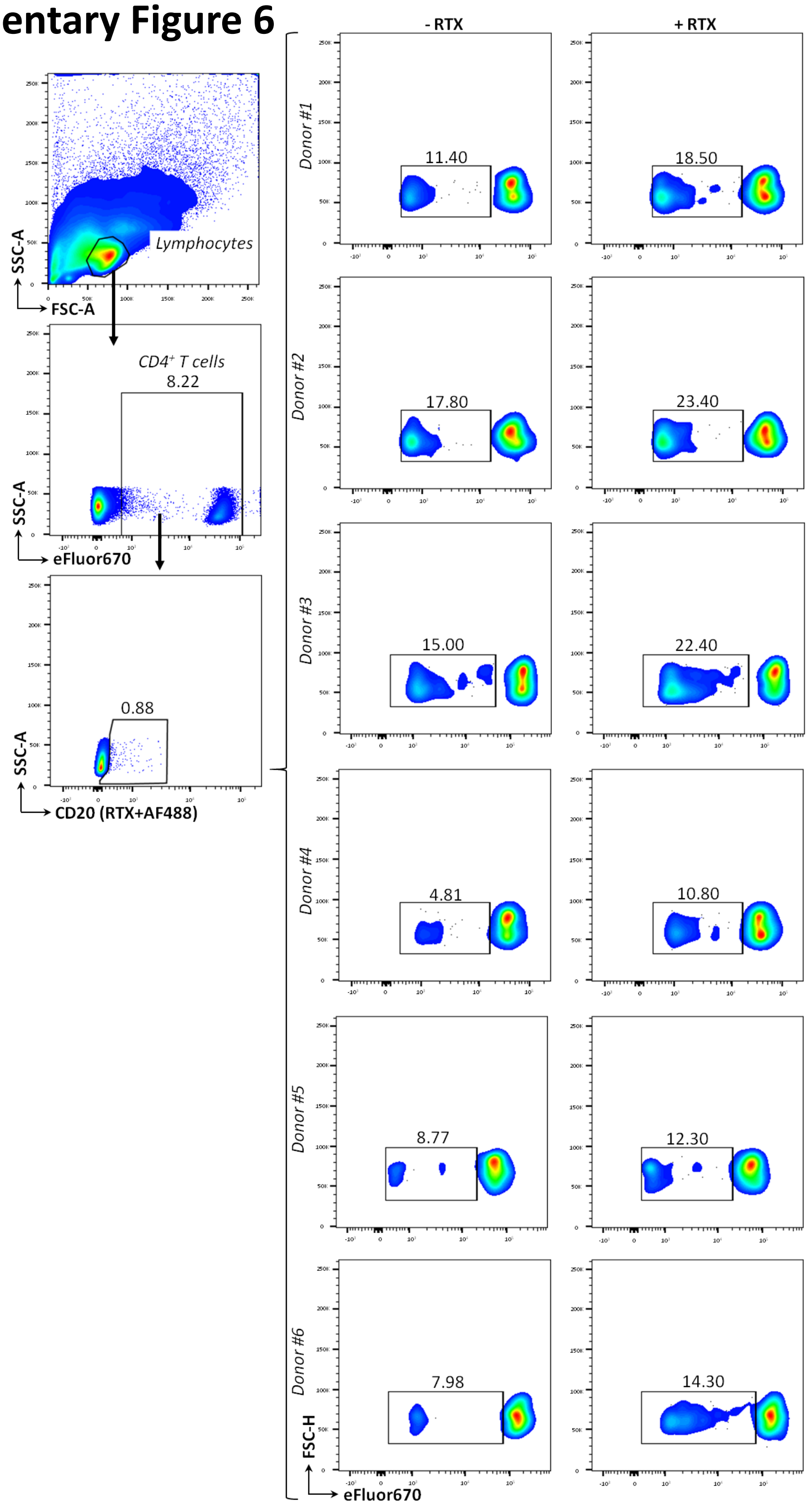

Supplementary Figure 6. Gating strategy for detection of ADCC in CD20^{dim} CD4^{+} T cells induced by Rituximab. Flow gating strategy used to identify cell cytotoxicity in the samples from 6 individuals are shown.

Supplementary Figure 7

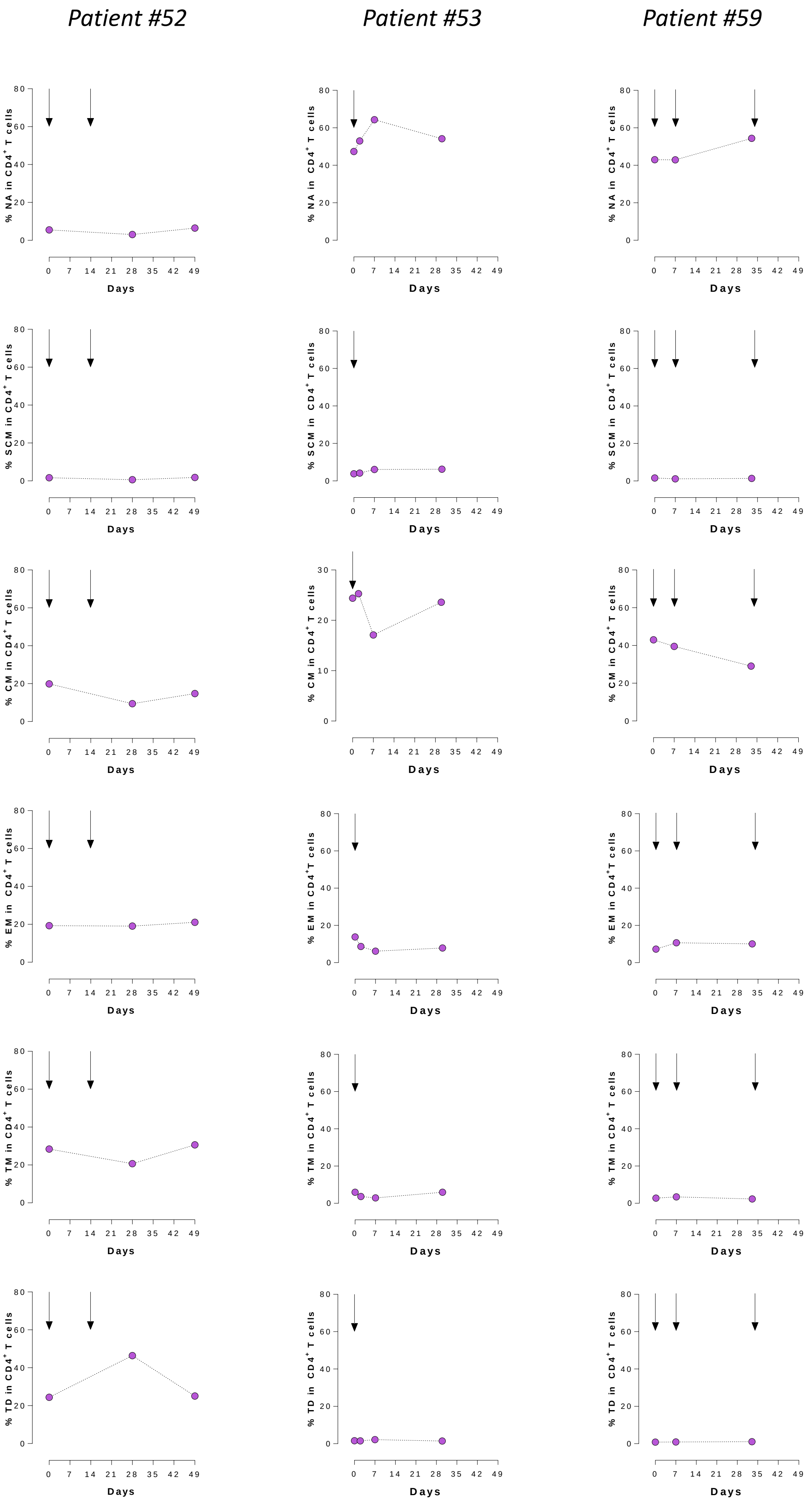

Supplementary Figure 7. Longitudinal changes in the proportion of $CD4^+$ T cell subsets after in vivo administration of Rituximab. Percentage of Naive ($CCR7^+$, $CD45RO^-$, $CD27^+$, $CD95^-$), Memory Stem cells ($CCR7^+$, $CD45RO^-$, $CD27^+$, $CD95^+$); Central memory ($CCR7^+$, $CD45RO^+$)Effector memory ($CCR7^-$, $CD45RO^+$, $CD27^-$), Transitional memory ($CCR7^-$, $CD45RO^+$, $CD27^+$); and Terminally differentiated ($CCR7^-$, $CD45RO^-$) in $CD4^+$ T cells are represented for patients, #52, #53 and #59. Flow gating strategy is shown in Figure 5. Data underlying this Figure is provided as Source Data file.

Supplementary Figure 8

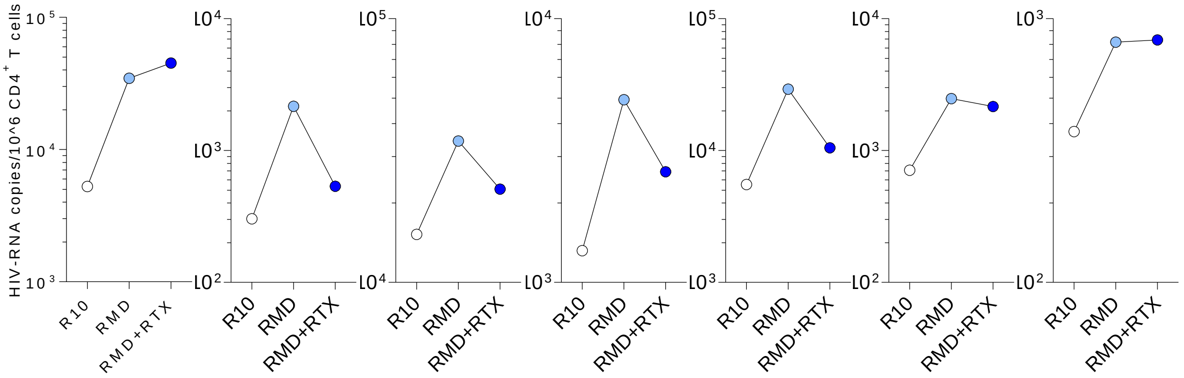

Supplementary Figure 8. Depletion of HIV RNA expressing cells by Rituximab after LRA treatment. Unfractionated PBMCs from ART-suppressed patients were treated with latently reversal agents (LRAs) romidepsin for 24h, and with Rituximab for 48h HIV RNA expression was measured by qPCR. The individual data for the HIV RNA quantification before and after viral reactivation with the addition of a control antibody or Rituximab in responder patients are shown. Patients #23 and 42-47. Data underlying this Figure is provided as Source Data file.

Supplementary Figure 9

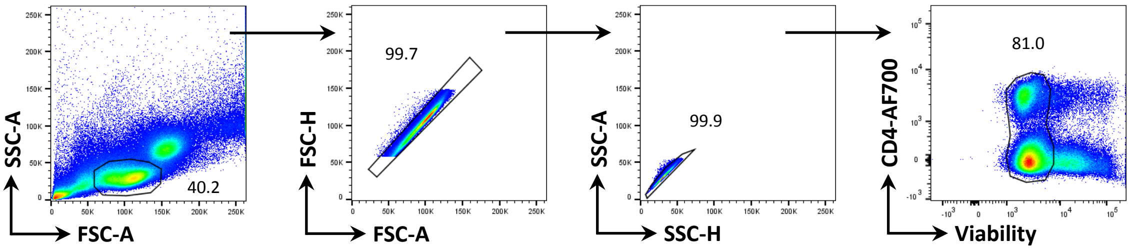

Supplementary Figure 9. Sequential flow gating strategy used in Figures 1, 2, 5, 6 and 7, and Supplemental Figures 1, 2, 3, 4, 5 and 7.
