# Supplementary material for: Expression of CD20 after viral reactivation renders HIV-reservoir cells susceptible to Rituximab
Source: Nat Commun. 2019 Aug 16;10:3705. doi: 10.1038/s41467-019-11556-4 (PMC6697690; doi:10.1038/s41467-019-11556-4)
Supplement: Supplementary file 3 — Reporting Summary [file 41467_2019_11556_MOESM3_ESM.pdf]

nature research

Corresponding author(s): DBPR NCOMMS-18-21646A-Z

Last updated by author(s): 16/07/2018

Reporting Summary

Nature Research wishes to improve the reproducibility of the work that we publish. This form provides structure for consistency and transparency in reporting. For further information on Nature Research policies, see Authors & Referees and the Editorial Policy Checklist.

Statistics

For all statistical analyses, confirm that the following items are present in the figure legend, table legend, main text, or Methods section.

n/a	Confirmed	
☐	☒	The exact sample size (*n*) for each experimental group/condition, given as a discrete number and unit of measurement
☐	☒	A statement on whether measurements were taken from distinct samples or whether the same sample was measured repeatedly
☐	☒	The statistical test(s) used AND whether they are one- or two-sided<br>*Only common tests should be described solely by name; describe more complex techniques in the Methods section.*
☒	☐	A description of all covariates tested
☐	☒	A description of any assumptions or corrections, such as tests of normality and adjustment for multiple comparisons
☐	☒	A full description of the statistical parameters including central tendency (e.g. means) or other basic estimates (e.g. regression coefficient) AND variation (e.g. standard deviation) or associated estimates of uncertainty (e.g. confidence intervals)
☒	☐	For null hypothesis testing, the test statistic (e.g. *F*, *t*, *r*) with confidence intervals, effect sizes, degrees of freedom and *P* value noted<br>*Give P values as exact values whenever suitable.*
☒	☐	For Bayesian analysis, information on the choice of priors and Markov chain Monte Carlo settings
☒	☐	For hierarchical and complex designs, identification of the appropriate level for tests and full reporting of outcomes
☒	☐	Estimates of effect sizes (e.g. Cohen's *d*, Pearson's *r*), indicating how they were calculated

Our web collection on statistics for biologists contains articles on many of the points above.

Software and code

Policy information about availability of computer code

Data collection	No software used.
Data analysis	We used GraphPad Prism. For flow cytometry analysis we used FlowJo V10. For flow imaging analysis IDEAS. For microscopy images analysis ImageJ.

For manuscripts utilizing custom algorithms or software that are central to the research but not yet described in published literature, software must be made available to editors/reviewers. We strongly encourage code deposition in a community repository (e.g. GitHub). See the Nature Research guidelines for submitting code & software for further information.

Data

Policy information about availability of data

All manuscripts must include a data availability statement. This statement should provide the following information, where applicable:

- Accession codes, unique identifiers, or web links for publicly available datasets
- A list of figures that have associated raw data
- A description of any restrictions on data availability

There are no restriction for any materials used in this study.

Field-specific reporting

Please select the one below that is the best fit for your research. If you are not sure, read the appropriate sections before making your selection.

☒ Life sciences ☐ Behavioural & social sciences ☐ Ecological, evolutionary & environmental sciences

Life sciences study design

All studies must disclose on these points even when the disclosure is negative.

Sample size	No sample-size calculations were performed. Sample size was determined to be adequate based on the magnitude and consistency of measurable differences between groups.
Data exclusions	No data were excluded.
Replication	Replicate experiments were successful.
Randomization	Randomization was not relevant to this study. Participants were chosen based on having an undetectable plasma HIV-RNA (< 50copies/ml) level or detectable plasma HIV-RNA (> 50copies/ml).
Blinding	Blinding was not relevant to this study because no bias could be made by the subject or the tester in the experiments performed.

Reporting for specific materials, systems and methods

We require information from authors about some types of materials, experimental systems and methods used in many studies. Here, indicate whether each material, system or method listed is relevant to your study. If you are not sure if a list item applies to your research, read the appropriate section before selecting a response.

Materials & experimental systems

n/a	Involved in the study	
☐	☒	Antibodies
☐	☒	Eukaryotic cell lines
☒	☐	Palaeontology
☒	☐	Animals and other organisms
☐	☒	Human research participants
☒	☐	Clinical data

Methods

n/a	Involved in the study	
☒	☐	ChIP-seq
☐	☒	Flow cytometry
☒	☐	MRI-based neuroimaging

Antibodies

Antibodies used	All antibodies used in this study are detailed in material and methods section.
Validation	All antibodies are commercially available and were commercially validated.

Eukaryotic cell lines

Policy information about cell lines

Cell line source(s)	TZM-bl (NIH AIDS Reagent Program) and 293T (ATCC, CRL-3216)
Authentication	None of the cell lines used were authenticated
Mycoplasma contamination	Cell lines used not tested for mycoplasma contamination
Commonly misidentified lines (See ICLAC register)	Not applicable

Human research participants

Policy information about studies involving human research participants

Population characteristics	All HIV+ patients were divided into two cohorts: ART-treated patients with undetectable viral load (< 50copies HIV-RNA/ml plasma), or patients with detectable viral load (> 50copies HIV-RNA/ml plasma).
Recruitment	Patients were recruited on opportunity for existing pre-established cohorts. Coded blood samples were obtained for these studies in questioin.
Ethics oversight	PBMCs from HIV-1-infected patients were obtained from the HIV unit of the Hospital Universitari Vall d'Hebron in Barcelona, Spain and from the Clinic Hospital in Barcelona. Study protocols were approved by the Comitè d'Ètica d'Investigació Clínica (Institutional Review Board numbers 39-2016 and 196-2015) of the Hospital Universitari Vall d'Hebron, and the Clinic Hospital,

Barcelona, Spain. Samples were obtained from adults, all of whom provided written informed consent, and were prospectively collected and cryopreserved in the Biobanc (register number C.0003590).

Note that full information on the approval of the study protocol must also be provided in the manuscript.

Flow Cytometry

Plots

Confirm that:

☒ The axis labels state the marker and fluorochrome used (e.g. CD4-FITC).

☒ The axis scales are clearly visible. Include numbers along axes only for bottom left plot of group (a 'group' is an analysis of identical markers).

☒ All plots are contour plots with outliers or pseudocolor plots.

☒ A numerical value for number of cells or percentage (with statistics) is provided.

Methodology

Sample preparation	PBMC were isolated by Ficoll density gradient centrifugation and cryopreserved. Thawed PBMC were pretreated with appropiated protocol (indicated in material and methods section) or immediately stained with indicated antibodies, then stained with viability dye and resuspended either in 1X PBS-5%FBS for sorting or in 2% paraformaldehyde for acquisition on an analyzer.
Instrument	BD FACS Fortessa analyzer, BD FACS Aria sorter, and an Amnis® ImageStreamx.
Software	FACS Diva for data collection. FlowJo and IDEAS software.
Cell population abundance	Purity was determined by running a purity check of the sorted populations after the sort was completed.
Gating strategy	All samples were initially gated using forward scatter and side scatter to identify events corresponding to cells, and then using forward scatter height vs. area to enrich for single cells, next alive cells were selected by negativity for viability dye. The follow gaiting steps are presented in principal and supplementary figures.

☒ Tick this box to confirm that a figure exemplifying the gating strategy is provided in the Supplementary Information.
